# High-Temperature Wear Performance of hBN-Added Ni-W Composites Produced from Combustion-Synthesized Powders

**DOI:** 10.3390/ma15031252

**Published:** 2022-02-08

**Authors:** Rahul Kumar, Sofiya Aydinyan, Roman Ivanov, Le Liu, Maksim Antonov, Irina Hussainova

**Affiliations:** 1 Department of Mechanical & Industrial Engineering, Tallinn University of Technology, 19086 Tallinn, Estonia; rahul.kumar@taltech.ee (R.K.); sofiya.aydinyan@taltech.ee (S.A.); roman.ivanov@taltech.ee (R.I.); le.liu@taltech.ee (L.L.); maksim.antonov@taltech.ee (M.A.); 2 A.B. Nalbandyan Institute of Chemical Physics NAS RA, P. Sevak 5/2, Yerevan 0014, Armenia

**Keywords:** self-propagating high-temperature synthesis, spark plasma sintering, sliding wear, high temperature, friction, Ni-W, hBN

## Abstract

This work reports on the spark plasma sintering (SPS) of self-propagating high-temperature-synthesis (SHS)-derived Ni-W and Ni-W-2wt%hBN (4:1 molar ratio of metals) powders. The synthesis was carried out from a mixture of NiO and WO_3_ using Mg + C combined reducers through a thermo-kinetic coupling approach. Experiments performed in the thermodynamically optimal area demonstrated the high sensitivity of combustion parameters and product phase composition to the amount of reducers and hBN powder. The powder precursors with and without the addition of hBN were consolidated using SPS at a temperature and pressure of 1300 °C and 50 MPa, respectively, followed by a thorough phase and microstructural characterization of the obtained specimens. SHS-derived powders comprised the nano-sized agglomerates and were characterized by a high sinterability. The specimens of >95% density were subjected to ball-on-plate dry sliding wear tests at a sliding speed of 0.1 ms^−1^ and a distance of 1000 m utilizing an alumina ball of 10 mm in diameter under a 15 N normal load. The tests were performed at a temperature of 800 °C. A significant improvement in wear behavior was demonstrated for SHS-processed composites in comparison with their counterparts produced via conventional high-energy ball milling technique owing to the phenomena of ‘micro-polishing’, cyclic ‘self-healing’ and fatigue. However, the decisive effect of hBN addition in imparting lubrication during an HT wear test was not confirmed.

## 1. Introduction

Mechanical, chemical or electro-chemical wear is a major concern in industries such as mining, material processing, electrical, chemical and surface engineering. The material damage by wear in such areas is sometimes a simultaneous approach of more than one wear mode (e.g., mechanical, corrosion or oxidation). Furthermore, in applications involving high temperatures (HTs), such as hot forming or forging, the wear mechanisms can be dramatically changed, resulting in a catastrophic failure and significant downtime. The incorporation of refractory metals, such as W, Mo, Ni and Cu in the transition metals, to enhance mechanical, tribological and corrosion properties, in conjunction with the preservation of thermal and electrical properties, is gaining popularity. In this respect, nickel-tungsten (Ni-W) alloys have shown fair potential for enhanced surface protection in electronics and mechanical industries; as high-temperature substrates for superconductors, barriers or capping layers in micro-electromechanical circuits; as catalysts for hydrogen evolution processes, etc., in comparison with elemental Ni [1]. Moreover, owing to its environmentally friendly nature, Ni-W alloys (composite or coating) can replace electroplated hard chromium or cadmium coatings in the areas of wear and corrosion protection, which are considered environmentally hazardous in the EU directives [2]. In recent years, the main fabrication techniques used for Ni-W alloys involve electrodeposition and mechanical alloying [3]. Alternative methods include the joint reduction of Ni and W oxides under heating in H_2_ or CO [4,5]. Nevertheless, due to large differences in their melting points (T_m(Ni)_ = 1455 °C, T_m(W)_ = 3422 °C), lattice structures and possibilities of various phase (intermetallics, solid solutions) formation, it is difficult to tailor the phase composition and achieve homogeneity in the alloy. Furthermore, electrochemical deposition of Ni-W results in a high amount of developed residual stress, which produces crack development [6,7]. Ni-W compositions with a moderate tungsten content (40 wt% vs. conventionally examined 15 wt% of W) are more complicated to deal with in terms of both synthesis and processing. To address this major challenge, there is a need to develop novel techniques aimed at the preparation of Ni-W composite powder that ensures a homogeneous distribution of phase and microstructure, along with improved mechanical and tribological characteristics.

Combustion synthesis (CS) fundamentally contributes to the field of green process intensification and has already demonstrated the ability to develop an extensive range of powders, near net shape products from ceramics, intermetallic, composites and multifunctional materials [8]. The intrinsic ‘green’ characteristics of CS or SHS are mainly related to the energy consumption arising exceptionally from the harnessing of heat produced during the reaction between reactants, the extremely reduced reaction time and sustainability. CS or self-propagating high-temperature synthesis (SHS) is an easy and inexpensive technique with tunable thermal settings for the fabrication of Ni-W (1:1 molar or 24:76 mass ratio) composite powder from a NiO-WO_3_-Mg-C powder mixture [9]. In previous studies, SHS was shown to deliver powders of Mo-Cu and W-Cu in the nano-to-submicron size range using a similar pathway [10,11,12]. It was earlier reported [13] that the incorporation of W into Ni matrix results in grain refinement at micro and nano levels promoted properties such as hardness and wear; however, a higher amount of W induced a decrease in oxidation stability of the sintered materials.

Hexagonal boron nitride (hBN) is a laminar solid with an easily sheared layered structure that is reported to offer a friction-reducing property [14]. Moreover, due to its high oxidation resistance (~1000 °C), good thermal conductivity and chemical inertness, it is often utilized in HT wear applications. A Ni-hBN electrodeposited coating to efficiently improve hardness, wear and corrosion resistance is reported in [15]. However, the incorporation of hBN in a Ni-W system to acquire better mechanical performance was conveyed by very few studies [16,17]. Moreover, studies on Ni-W-hBN systems fabricated via combustion synthesis technique are absent to the best of our knowledge. The current work reports the fabrication of Ni-W and Ni-W-hBN systems with moderate tungsten content (40 wt%) through a more efficient method of SHS. Phase and structure evolution in Ni-W and Ni-W-hBN systems, along with homogeneous distribution of phases (metals, intermetallics, solid solutions), are essential issues regarding the synthesis and sintering to overcome phase segregation, under-sintered areas and cracking. The adiabatic combustion temperature and equilibrium phase composition of the products were first evaluated using thermodynamic modeling for the 4NiO-WO_3_-Mg-C mixture and combustion peculiarities were disclosed. The possibility of one-stage preparation of 4Ni-W and 4Ni-W-hBN composite powders (hereinafter Ni-W and Ni-W-hBN) from oxide precursors by using the effective energy-efficient way (by using the thermo-kinetic coupling approach) at moderate temperature conditions was explored. The SHS-derived Ni-W and Ni-W-hBN composite powders were further put through densification via spark plasma sintering (SPS). The SPS-derived specimens were assessed for their response to HT in an 800 °C tribological environment. The results are discussed in reference to the SPS-consolidated bulks from high-energy ball-milled (HEBM) Ni-W and Ni-W-hBN composites.

## 2. Experimental

### 2.1. Combustion Synthesis

The following precursors were used for the combustion experiments (via SHS): tungsten (VI) oxide (>99.8%, 10–20 μm, Alfa Aesar, Haverhill, MA, USA), nickel oxide (>99%, <44 μm, Alfa Aesar), magnesium powder (>99%, 150 μm, Alfa Aesar), graphite KS6 and hexagonal boron nitride powder (>99%, 20 μm). The initial powders were homogeneously mixed using a pestle in a ceramic mortar for 20 min, and 23.4 mm in diameter cylinder-shaped green bodies of 1.7–1.8 g/cm^3^ density and 40–45 mm height were prepared. The prepared cold compacts were then placed in a constant pressure reactor CPR-3L (Sapphire Co., Abovyan, Armenia). The chamber was closed, evacuated, purged with nitrogen (>99.98%) and filled to the intended pressure up to 0.4 MPa. Short heating (3 s) of the tungsten spiral attached to the top surface of the sample initiated the combustion wave propagation in the mixture under study. Combustion temperature and wave propagation velocity were registered via W/Re5-W/Re20 thermocouples that were 100 μm in diameter. The maximum combustion temperature (T_c_) was taken from the temperature profile. The U_c_ = L/t formula was used for the calculation of the combustion velocity, where the distance between two thermocouples is indicated by L and the time interval between signals from thermocouples is indicated by t. The standard error for the T_c_ measurement was ±20 °C and ±5% for U_c_. The reacted sample was then crushed to a powder, sieved through a sieve with a mesh size of 100 microns and exposed to acid leaching by a 10% HCl solution at 40 °C for 20 min to remove the magnesia by-product. The combustion product after leaching comprised <20 μm particles of bimodal morphology: agglomerates of glued nanoparticles and pre-sintered crystals in both mixtures with and without hBN.

### 2.2. High-Energy Ball Milling (HEBM)

Mechanical milling of Ni and W powder mixture was implemented in a high-energy ball mill (Emax, Retch GmbH, Haan, Germany) in 125 mL jars. Metal powders (60 wt% of nickel and 40 wt% of tungsten) were first dry mixed in a ceramic mortar. Wet milling in the presence of ethanol (75 vol% of the jar) was performed using 3 mm zirconia balls as a milling media with a powder-to-ball mass ratio of 1:1. The milling conditions were set as follows: 1000 rpm for 12 h using intervals of 15 min and a pause for 5 min. After milling, the mixture was sieved to detach the balls and dried at 50 °C to remove the ethanol.

### 2.3. Spark Plasma Sintering (SPS)

The combustion synthesized powders were consolidated via the spark plasma sintering technique (KCE-FCT HP D 10-GB, FCT Systeme GmbH, Frankenblick, Germany) with simultaneous application of 50 MPa pressure, up to 1300 °C (1050, 1150, 1200, 1300 °C) temperature and a high-density current within a vacuum for dwell times of 5, 10, 20 and 30 min. The SHS powder was poured into a graphite die of 20 mm inner diameter. Graphite sheets were placed between the punch and the powder. The hBN spray was utilized to hinder the graphite interaction with the tungsten. The heating rate was set to 100 °C/min. The thickness of the produced specimens was 3 mm.

### 2.4. Mechanical, Phase and Microstructural Characterization

The Archimedes method was used to measure the bulk density of the prepared SPS-derived composites (Mettler Toledo ME204, Greifensee, Switzerland), where distilled water was needed as an immersion medium. The theoretical density of the mixture was calculated using the rule of mixture (the densities of nickel and tungsten were taken as 8.90 g·cm^−3^ and 19.25 g·cm^−3^, respectively). According to the rule of mixture, the densities of the Ni-W and Ni-W-2wt%hBN composites were calculated as being 11.65 g·cm^−3^ and 10.69 g·cm^−3^, respectively.

Phase analysis was accomplished via X-ray diffraction (XRD) using a Philips X’Pert PRO diffractometer (40 mA, 40 kV, CuKα radiation, λ = 0.1542 nm, step size of 0.02°, PANalytical, Malvern Panalytical B.V., Almelo, The Netherlands) for both crashed powders (after SHS) and SPS specimens. The HighScore Plus software database was used to determine the XRD peaks (ICCD cross-referenced). The relative amounts of different phases in the composites were estimated through the Rietveld refinement method

A field-emission scanning electron microscope (FE-SEM, Zeiss Evo MA15, Oberkochen, Germany) equipped with an EDS detector was utilized. Samples were subjected to a hot conductive (resin) mounting, polishing and coating (not required for tribological testing) using a thin layer of Pt to provide sufficient conductivity. The fabricated specimens were polished (Phoenix 4000, Buehler) down to a 0.5 µm finish using water as a medium with 8-inch diamond grinding discs (DGD Terra, Buehler, Esslingen, Germany). The polishing was performed with a speed of 200 rev/min for 4 min for each grinding disc. The polished specimens were cleaned using acetone and ethanol for their further study.

The Vickers hardness tester (Indentec 5030 SKV, Stourbridge, West Midlands, UK) was applied to estimate the hardness of the bulk samples against the indentation load of 10 kg applied for 10 s. The average of at least 5 indentations is reported.

### 2.5. High-Temperature Wear Tests

**Figure 1:**
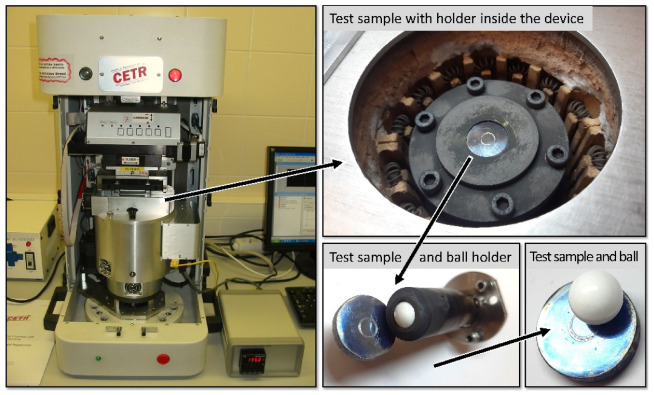
A universal tribo-test device (UMT-2) employed for the HT dry unidirectional sliding.

HT-800 °C wear tests were performed on a universal tribo-test device (CETR/Bruker, Billerica, MA, USA) in a dry unidirectional-sliding ball-on-plate arrangement (Figure 1) [18]. The counterbody was an Al_2_O_3_ ceramic ball (Redhill Precision, Prague, Czech Republic) with Ø10 mm, hardness HV10 ≈ 1450 and roughness Ra = 0.02 μm. The sample surface was cleaned with acetone and dried before the tests. The speed and distance of the sliding were set to 0.1 m·s^−1^ and 1000 m, respectively, with an applied load of 15 N. The duration of the test was 165 min. The choice of the load was made upon optimization to generate measurable wear. A heating rate of 6 °C/min was decided to avoid unwanted thermal shock for the materials and equipment under the test. The coefficient of friction (CoF) was recorded during sliding. The generated wear tracks were inspected through SEM and XRD, and were calculated using a 3D optical profilometer (Bruker Contour GT-K0+, USA) to determine the volume of the wear scar. The results reported are an average of at least five tests. The tests were conducted in ambient air.

## 3. Results and Discussion

### 3.1. Thermodynamic Modeling and Combustion Synthesis

**Figure 2:**
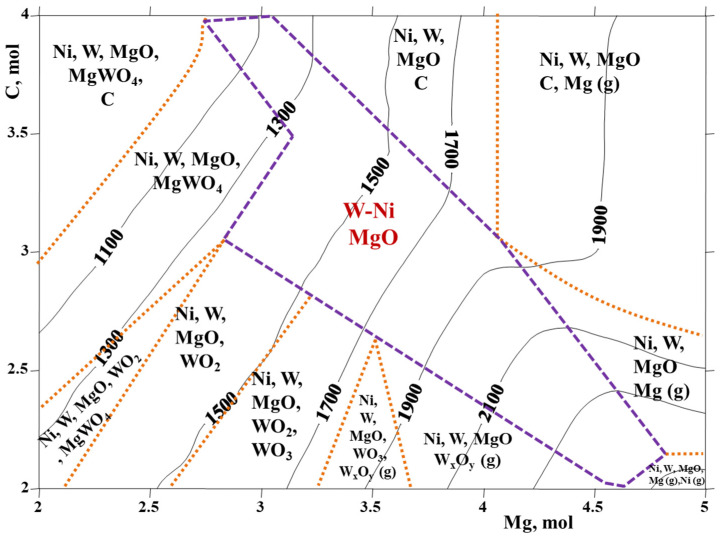
Thermodynamic modeling of the 4NiO-WO_3_–yMg-xC system (P_N2_ = 0.4 MPa).

Thermodynamic considerations implemented for the 4NiO-WO_3_-yMg-xC system allowed for modeling the optimal parameters for the combined and entire reduction of the corresponding metals’ oxides using the ‘ISMAN-THERMO’ software package. The system was designed to obtain Ni-W alloys with a molar composition ratio of Ni:W = 4:1. The thermodynamic design enabled evaluating the combustion temperature (T_ad_) in adiabatic conditions and the equilibrium phase composition of combustion products in the multielement system [8]. As a result, a diagram of phases with corresponding adiabatic temperatures (in °C) was created depending on the reducers’ (Mg, C) contents (in moles) (Figure 2). Various compositional areas were identified according to the composition of the initial mixture, namely, the amounts of Mg and C. According to the diagram, a wide compositional range corresponded to the formation of the target product (magnesium from 2.7 to 4.8 moles and carbon from 2 to 4 moles) (marked in Figure 2) at a T_ad_ of 1000–2200 °C. Compared with the Ni-W 1:1 mixture (where magnesium ranged from 1.7 to 2.2 mol and carbon from 1.6 to 2.3 mol), a thermodynamically wider area of the 4Ni-W desired product was expected. It is to be noted that the thermodynamic calculations were performed excluding the possibility of the formation of tungsten carbides (WC, W_2_C) by considering the kinetic inhibition of carbide formation processes at moderate temperatures over a short duration. Furthermore, a necessity of minimum temperature, i.e., 1700 °C, to form tungsten carbides upon a short interaction duration of less than a few seconds (compared to the characteristic duration of combustion wave propagation) was also reported in [19,20]. However, during a slow combustion process (0.1–0.2 mm/s), the formation of some amount of tungsten carbides is mostly inevitable.

According to the thermodynamic calculations, within the entire range of the reducers’ quantity, the main gaseous products were CO and CO_2_. Their ratios depended on the combustion temperature and shifted to CO formation at a higher amount of Mg (>3.2 mol), a lower amount of carbon (<3 mol) and a higher temperature. Moreover, at high temperatures, magnesium (Mg), WO_2_ and (WO_3_)_n_ were also evident.

**Figure 3:**
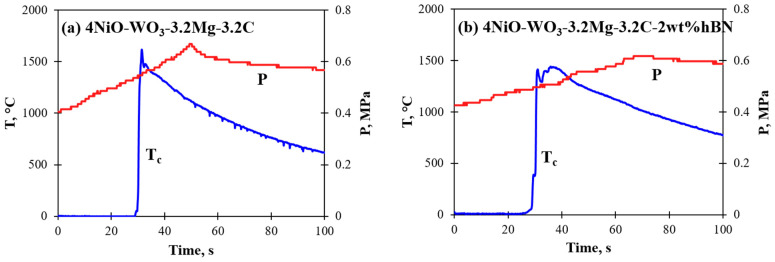
Combustion reaction thermograms of (**a**) 4NiO-WO_3_-3.2Mg-3.2C and (**b**) 4NiO-WO_3_-3.2Mg-3.2C-2wt%hBN mixtures.

To achieve the combined and accomplished reduction of oxides, as well as to avoid the evaporation of magnesium and various tungsten oxides, a pressure of 0.4 MPa was found to be optimal. To avoid nickel melting, the experiments were performed only in the lower temperature range (1100–1500 °C) of the optimal range (marked as violet borders on the diagram) for different contents of magnesium and carbon. During the synthesis, an increase in combustion temperature was observed with a decrease in carbon amount and increasing Mg amount, conditioned by the decrease in the portion of low caloric carbothermic reactions (Ni + C and/or WO_3_ + C) and an increase in the high caloric mixture (WO_3_ + Mg). Combustion velocity, heating and cooling rates were also increased. Combustion thermograms with the pressure change during the combustion reaction of the optimal mixtures are presented in Figure 3. The addition of hBN (2 wt% according to the mass of the final Ni-W product) decreased the combustion parameters by 200 °C and played the role of an inert diluent.

The combustion parameters of the reactive mixtures (Figure 3a,b) are presented below in Table 1, mainly denoting the changes in T_c_ and U_c_, which were sensitive to the changes in the reducers’ amount and hBN powder. In addition, mass and pressure changes were observed due to CO/CO_2_ formation. The combustion temperature was about 150–200 °C higher than the adiabatic temperature conditioned by the exothermic formation of the Ni-W intermetallic phases/solid solutions, in contrast to thermodynamic consideration of only the formation of individual metals.

XRD analysis of the combustion products revealed the presence of trace amounts of tungsten carbides and partially reduced WO_2_ oxide, together with Ni-W, W-Ni and MgO phases in the 4NiO-WO_3_-3.2Mg-3.2C and 4NiO-WO_3_-3.2Mg-3.2C-2wt%hBN mixtures. A BCC-structured W-rich W_0.968_Ni_0.032_ intermetallic phase with Im-3m space group symmetry was detected at 2t = 40.28, 58.28, 73.2 and 87.04 (pure tungsten 2t = 39.966, d = 2.254 (hkl 210)). Broad lines of Ni-rich two phases with Ni_9_W_0.4_ and Ni_0.954_W_0.046_ phase compositions with FCC structures of Fm-3m space group symmetry were located at 2t = 44.24, 51.32, 75.04 and 91.68 (for pure nickel 2t = 43.97 (hkl 211), d = 2.0576). Further, a MgO periclase phase was successfully removed with a hot leaching procedure. The Scherer equation (d (nm) = kλ/βcosθ) was used for the assessment of the average crystallite size of the W-rich and Ni-rich phases. The W-rich phase had an average crystallite size of 84.62 nm, and the nickel-rich phase had an average crystallite size of 22–42 nm (as two different Ni_9_W_0.4_ and Ni_0.954_W_0.046_ phases were merged into one peak).

**Figure 4:**
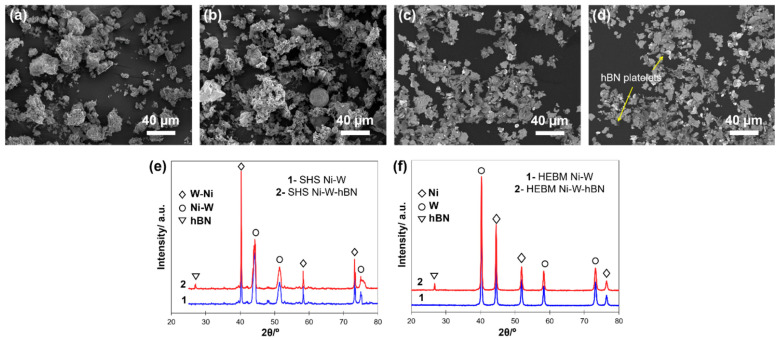
SEM images of powders mixtures of (**a**) SHS-derived Ni-W, (**b**) SHS-derived Ni-W-hBN, (**c**) HEBM Ni-W and (**d**) HEBM Ni-W-hBN, and XRD results of (**e**) SHS-derived powders and (**f**) HEBM powders.

Figure 4 shows the SEM and XRD of SHS- and high-energy ball milling (HEBM)-derived powders. The XRD patterns of the SHS-derived Ni-W powder (Figure 4e) showed broader peaks of Ni-W and W-Ni (compared with the elemental Ni and W, Figure 4f), confirming the partial dissolution (the formation of solid solution) of W in Ni and vice versa during the combustion synthesis. Apart from the phase dissolution, the SHS technique formed composite particles or agglomerates of Ni and W of bimodal morphology (Supplementary Figure S1).

Snowflake particles of up to 100 nm average size and spheroidal particulates of up to 300 nm in size and with well-defined grain boundaries were observed. Usually, such kinds of SHS-derived powders (pre-sintered agglomerates of nanosize entities) demonstrate an enhanced sintering ability due to high heating and cooling rates present in exothermic combustion reactions and a significant defect concentration that largely contributes to the mass transfer phenomena during the sintering.

### 3.2. Spark Plasma Sintering and Microstructural Analysis

SHS-derived and commercial powders were subsequently subjected to SPS at various temperatures (1050, 1150, 1200 and 1300 °C) at a pressure of 50 MPa with a dwell time of 5–30 min to bring out an optimal sintering condition with a maximum product density. It was found that the composites sintered at 1300 °C exhibited a relative density of >95%. SHS-processed composites sintered at 1300 °C and 50 MPa with a dwell time of 5 min and HEBM-processed composites sintered at 1100 °C and 50 MPa with a dwell time of 30 min achieved an improved relative density, as well as hardness. The influence of the processing technique and hBN inclusion on the density and hardness of the Ni-W (-hBN) sintered bulks (optimized at 1300 °C and 50 MPa—current work) is outlined in Table 2, along with values taken from the recently published literature reporting on Ni-W sintering. The SHS-processed composites in the current work show a significant rise in hardness and density owing to the developed phases and microstructure (discussed later).

**Figure 5:**
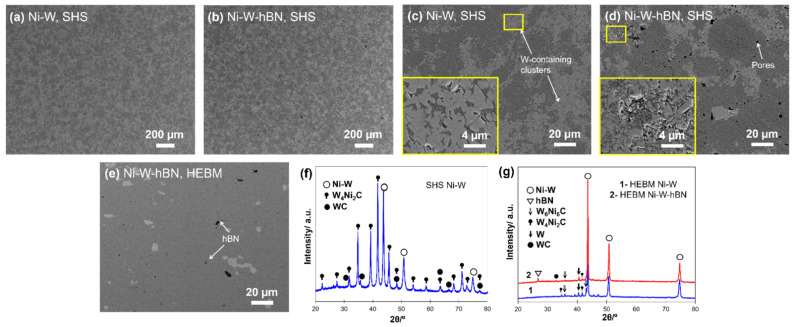
SEM images of SPS-derived composites (**a**,**c**) SHS-derived Ni-W, (**b**,**d**) SHS-derived Ni-W-hBN and (**e**) HEBM Ni-W-hBN and XRD results of (**f**) SHS SPS-derived bulk and (**g**) HEBM SPS-derived bulks.

Figure 5 shows the SEM and XRD results of the sintered composites produced from SHS, as well as commercial powders. Based on the XRD analysis (Figure 5f,g) of the composites, it can be stated that, apart from primary intermetallic NiW, the contained phases in the bulks were carbides, such as WC, W_6_Ni_6_C and W_4_Ni_2_C. The presence of carbides in the composites was due to the fact that graphite was used as a die, punch and covering sheet during the SPS, which might have resulted in carbon diffusion to the composites. In order to gain insight into this phenomenon, the cross-sections of composites were also analyzed using XRD, and unexpectedly, the presence of W_6_Ni_6_C, W_4_Ni_2_C and sometimes W_2_C phases were still evident. The formation of W_6_Ni_6_C and W_4_Ni_2_C at the cross-section (subsurface) was supported by the fact that it required the least amount of carbon (<0.8%) in comparison with WC or W_2_C, which was mostly seen at the surface. A similar result of carbon uptake throughout the volume of the sintered Ni-W compacts was reported by [20,25]. It was conveyed that nickel, unlike tungsten, does not form stable carbides; nevertheless, it provides a channel for carbon diffusion inside the compacts. This demonstrates the fact that the diffusion of carbon was deep (at least 1.5 mm) into the sintered compacts. SPS samples prepared from SHS powder comprised comparatively more intense peaks of W-C and W-Ni-C phases than that of HEBM ones (as well as sintered bulks reported in the literature [21,22,23,24,25]). This was conditioned by the enhanced reactivity of the SHS powder and inherently high defect concentration. Even with the presence of a negligible amount of carbon, the tungsten solid solution dissolved it, forming WC while simultaneously precipitating nickel and W-Ni-C without nickel precipitation. Limited peaks of residual W existed in the XRD results of the HEBM-processed composites, conveying its inability to be sintered using the lower sintering temperature. From the SEM images (Figure 5a,b) of the SHS SPS-derived samples’ surfaces, a homogeneous distribution of different phases was obvious with the presence of random pores. With the addition of hBN powder, the phase distribution did not present changes, but the number of pores increased (Figure 5b).

As per the SEM images with a higher magnification (Figure 5c–e), the two techniques demonstrated different microstructures. The bright whitish patches (columns in the magnified image) are believed to be the clusters of tungsten distributed in the Ni-rich matrix. Since it was difficult to differentiate between the W in the matrix and its carbides during the EDS analysis, the bright whitish clusters were anticipated to be enriched by tungsten, as well as its carbides. The grey matrix was a Ni-rich Ni-W solid solution. A homogeneous distribution of W clusters was evident in the Ni-W alloy phase processed via SHS.

An increase in the surface pores (Figure 5d,e) upon hBN addition was clearly visible in the respective composites, regardless of the fabrication method. A higher-magnification image showed the detachment of grains due to the reduced sinterability owing to hBN incorporation. It is possible that the hBN agglomerated, further growing the size of pores and decreasing the adhesion between grains, resulting in their detachment and easy falling out during the polishing stage.

All composites revealed a higher hardness in comparison to the pure Ni bulk (~110 HV). The improved hardness of the composites (more than 4 and 2 times for SHS and HEBM powder-processed bulk, respectively) is explained by a solid solution strengthening and the effective densification during sintering. The obtained values for SHS processed composites were consistent with the reported results of Ni-W systems (about 4.3 GPa) [21,22,23,24]. A larger margin in the hardness results between two processing techniques could be explained by the phenomena of solid solution strengthening. The formation of a considerable amount of ductile Ni-rich solution in HEBM-processed composites of Ni-W (~85.5%) was responsible for the relatively low hardness of the material. The in situ formation of carbides during the SPS allowed not only for successful sintering but also for enhanced performance. An increase in the hardness of sintered Ni-W bulks due to the presence of W-based carbides (mainly WC) was also reported in [21,22]. A slightly reduced hardness of hBN-containing composites in comparison to their counterparts was mainly due to the residual porosity of the sintered bulks (Figure 5d,e).

### 3.3. High-Temperature Wear Tests

**Figure 6:**
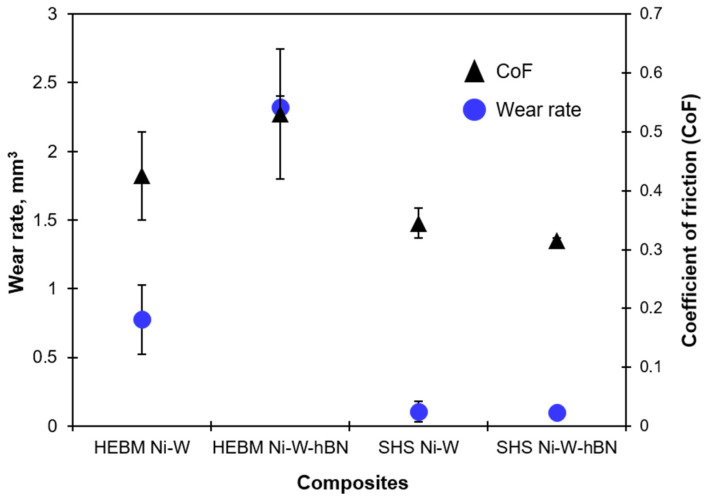
Wear rate and CoF of SPS-derived composites. The method of powder preparation is mentioned.

Figure 6 shows the CoF and wear rate of Ni-W and Ni-W-hBN composite produced from HEBM and SHSed powders. A decrease in friction and wear rate (up to 20 times for the wear of the hBN-containing composite) was noted for the SHS-processed composites in comparison to their HEBM-processed counterparts. However, since (1) the reduction in friction and wear rate was noted for both (with and without hBN) composites and (2) their respective values remained in a comparable range, it is hard to comment on the positive influence of hBN. Moreover, the hBN inclusion led to a considerable rise in the wear rate and CoF for the HEBM-processed samples. Similar behavior of composites containing hBN was reported in [26,27,28]. Nevertheless, the friction coefficient values of SHS-processed composites were seen to be up to 40% lower than that of the HEBM-processed samples, signifying a positive effect of the SHS technique on Ni-W composite preparation.

**Figure 7:**
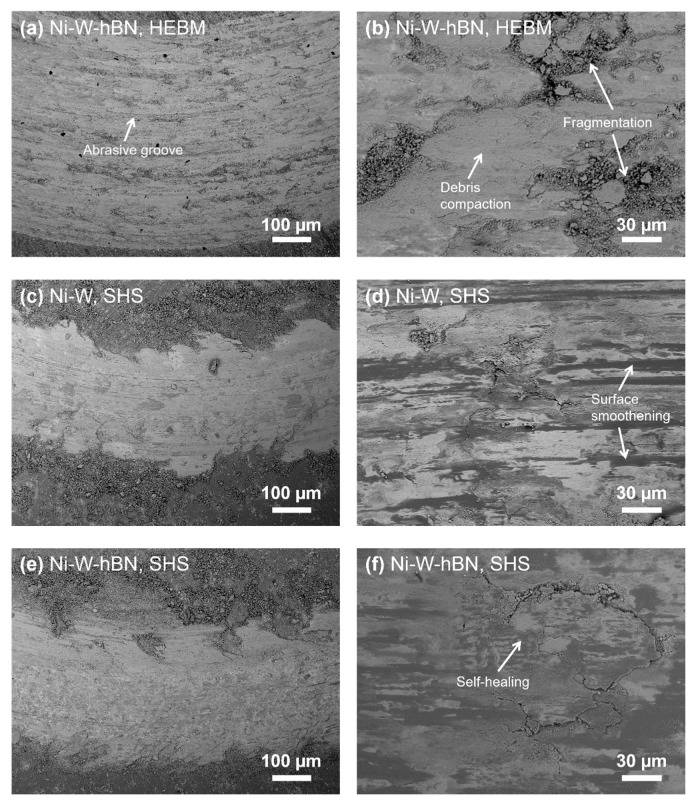
SEM images with two magnifications of worn surfaces of SPS-derived composites (**a**,**b**) HEBM Ni-W-hBN, (**c**,**d**) SHS-derived Ni-W and (**e**,**f**) SHS-derived Ni-W-hBN.

From the micrographs of the wear tracks, as shown in Figure 7, the high severity of the abrasive grooves and surface delamination is clearly evident on the surface of HEBM-processed Ni-W-hBN specimen (Figure 7a). Figure 7b displays a compacted layer of adhered wear debris, indicating the prevalence of adhesive wear. Such adhered debris can greatly increase wear resistance via the formation of an effective tribolayer or healing the defected areas [29,30]. However, during successive sliding passes, the tribolayer underwent fragmentation, resulting in a loss of the tribolayer protection. A similar wear behavior was demonstrated by the HEBM-processed Ni-W composite. Therefore, it was realized that abrasion and adhesion were the main wear mechanisms for the HEBM-processed composites. In contrast, the SHS-processed composites showed a considerably reduced severity of wear and demonstrated a more compact adhered tribolayer on the surface.

Compaction and smoothening (or micro-polishing) of the surface during consecutive sliding passes were dominant for SHS-processed composites. It is possible that some debris that was generated during the test were relocated into the voids and grooves, resulting in a ‘debris-entrapment’ and compaction phenomena during sliding that can be termed as ‘self-healing’ [14,31]. During a relatively long wear test of 165 min, the fatigue-induced delamination of debris may occur [18]. The SEM images of SHS-processed composites (Figure 7d,f) demonstrated traces of surface fatigue cracks showing material removal from the surface. Here, the cyclic delamination and repair of the tribolayer, along with mild abrasions, were considered as the main wear mechanisms.

**Figure 8:**
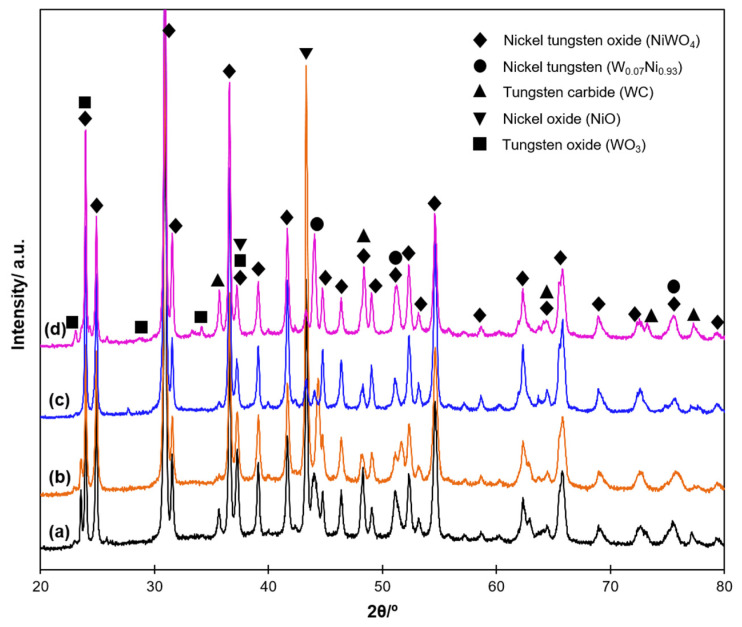
XRD analysis of the wear track of the SPS-derived composites (**a**) HEBM Ni-W, (**b**) HEBM Ni-W-hBN, (**c**) SHS-derived Ni-W and (**d**) SHSed Ni-W-hBN.

The XRD patterns of the wear tracks for all composites, presented in Figure 8, displayed the sharp peaks of Ni-W oxides, along with an insignificant amount of the broad peaks of NiO, WO_3_ and WC. Since the oxides were formed during the HT wear test, as their presence was not detected after the SPS, it can be concluded that the compaction of generated oxides or oxide debris took part in the tribolayer formation.

## 4. Conclusions

A thermodynamic model of a 4NiO-WO_3_-yMg-xC system was used to estimate the optimal criteria for obtaining Ni-W alloys with a molar composition ratio of Ni:W = 4:1. Experiments conducted according to the model indicated the influence of the reducer’s amount and hBN addition on the combustion parameters and product phase composition. XRD analysis of combustion products revealed the presence of BCC-structured W-rich and FCC-structured Ni-rich phase compositions. The combustion synthesized (via SHS) powders demonstrated a ‘snow-flake structure’, encompassing pre-sintered agglomerates of nano-sized entities.

SHS and HEBM powders of Ni-W and Ni-W-hBN were successfully consolidated with full density using SPS. The SHS-derived ‘snow-flake structured’ powders demonstrated a high sinterability (5 min vs. 30 min for HEBM powders), commendable hardness and a superior high-temperature tribological performance (800 °C). In comparison to their counterparts, SHS-processed composites displayed a lower CoF and wear rate owing to the phenomena of ‘micro-polishing’ and cyclic ‘self-healing’ of the surface during sliding. However, the role of hBN in imparting lubrication during the HT wear test was not confirmed.

## Figures and Tables

**Table 1:** Optimized combustion parameters of the mixtures (a) 4NiO-WO_3_-3.2Mg-3.2C and (b) 4NiO-WO_3_-3.2Mg-3.2C-2wt%hBN (P = 0.4 MPa).

No.	T_c_ (T_ad_), °C	U_c_, mm/s	U_heating_, °C/s	U_cooling_, °C/s	∆m_exp_, %	∆m_theor_, %	CO/CO_2_	∆P, atm
a	1610 ± 20 (1450)	0.76 ± 0.04	870 ± 90	14 ± 2	23.7 ± 2.1	15.4	2.6/0.6	2.2 ± 0.1
b	1420 ± 20 (1280)	0.63 ± 0.03	700 ± 70	6 ± 1	19.7 ± 1.8	15.3	3.2/0.3	2.1 ± 0.1

**Table 2:** Comparison of fabrication technique, relative density and hardness of Ni-W composites.

Composite	Fabrication Technique	Relative Density (%)	Hardness (HV)
Ni-30 wt.% W [21]	Mechanical alloying (MA) + pressureless sintering	97.8	367 ± 21 *
Ni-30 wt.% W [22]	MA + SPS	96.7	430 ± 10 *
Ni-30 wt.% W-hBN [22]	MA + SPS	95.3	410 ± 12 *
Ni-40 wt.% W [23]	MA + pressureless sintering	91.1	425 ± 05 *
Ni-35 wt.% W [24]	Blending + SPS	-	321 ± 26 *
Ni-50 wt.% W [24]	Blending + SPS	-	414 ± 34 *
Ni-40 wt.% W (current work)	SHS + SPS	98.6	460 ± 31
Ni-40 wt.% W-hBN (current work)	SHS + SPS	95.8	437 ± 12
Ni-40 wt.% W (current work)	HEBM + SPS	98.9	284 ± 10
Ni-40 wt.% W-hBN (current work)	HEBM + SPS	97.4	271 ± 10

* relates to microhardness, load 100–200 g. Current work involved a load of 10 kg.

## Data Availability

Data available on request.

## Supplementary Materials

The following are available online at https://www.mdpi.com/article/10.3390/ma15031252/s1, Figure S1: XRD patterns (a); and SEM image (b) of combustion product of mixtures 4NiO-WO_3_-3.2Mg-3.2C-2wt%h-BN before acid leaching.
