# Supplementary material for: High-Temperature Wear Performance of hBN-Added Ni-W Composites Produced from Combustion-Synthesized Powders
Source: Materials (Basel). 2022 Feb 8;15(3):1252. doi: 10.3390/ma15031252 (PMC8839063; doi:10.3390/ma15031252)
Supplement: Supplementary file 1 [file materials-15-01252-s001.zip › materials-1557254-supplementary.pdf]

materials

MDPI

Supplementary Materials

High-Temperature Wear Performance of hBN-Added Ni-W Composites Produced from Combustion-Synthesized Powders

Rahul Kumar, Sofiya Aydinyan, Roman Ivanov, Le Liu, Maksim Antonov and Irina Hussainova *

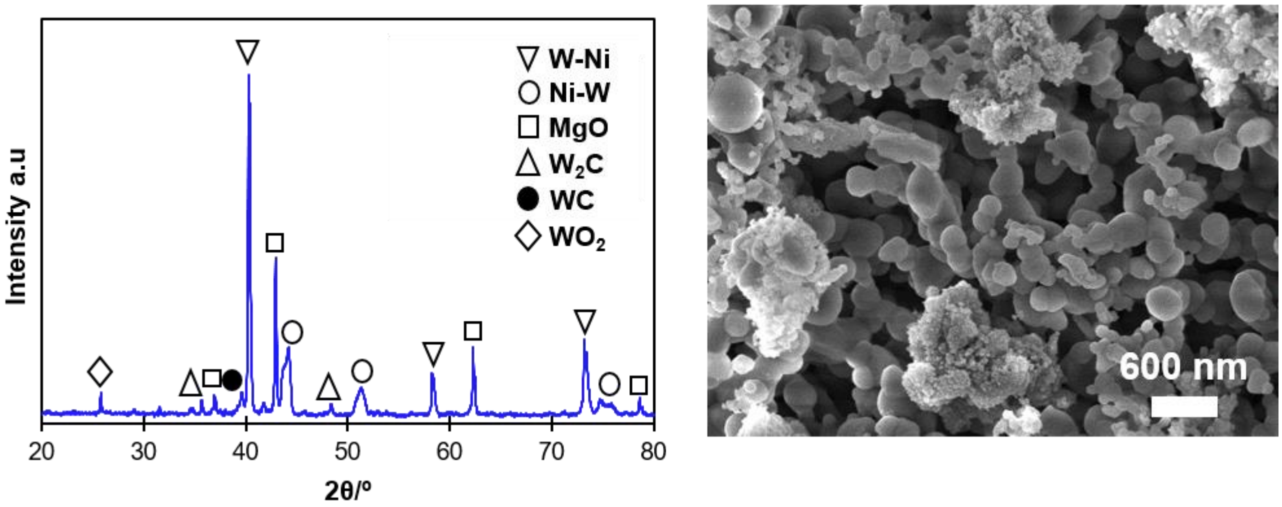

(**a**) (**b**)

Figure S1. XRD patterns (**a**); and SEM image (**b**) of combustion product of mixtures 4NiO-WO_3-3.2Mg-3.2C-2wt%h-BN before acid leaching.
